# *Bixa orellana* L. (Bixaceae) and *Dysphania ambrosioides* (L.) Mosyakin & Clemants (Amaranthaceae) Essential Oils Formulated in Nanocochleates against *Leishmania amazonensis*

**DOI:** 10.3390/molecules24234222

**Published:** 2019-11-20

**Authors:** Laura Machín, Beatriz Tamargo, Abel Piñón, Regla C. Atíes, Ramón Scull, William N. Setzer, Lianet Monzote

**Affiliations:** 1 Department of Pharmacy, Institute of Pharmacy and Food, Havana University, Havana 17100, Cuba; laura@ifal.uh.cu (L.M.); raties@ifal.uh.cu (R.C.A.); rscull@ifal.uh.cu (R.S.); 2 Department of Physiological Science, Latin American School of Medical Sciences, Havana 11300, Cuba; btamargo@infomed.sld.cu; 3 Department of Parasitology, Institute of Tropical Medicine Pedro Kourí, Havana 17100, Cuba; abelpt@ipk.sld.cu; 4 Department of Chemistry, University of Alabama in Huntsville, Huntsville, AL 35899, USA; 5 Aromatic Plant Research Center, 230 N 1200 E, Suite 100, Lehi, UT 84043, USA; 6 Research Network: Natural Products against Neglected Diseases (ResNet NPND)

**Keywords:** *Bixa orellana*, *Chenopodium ambrosioides*, essential oil, *Leishmania amazonensis*, nanocochleate

## Abstract

Leishmaniasis is a group of neglected tropical diseases caused by protozoan parasites of the *Leishmania* genus. The absence of effective vaccines and the limitations of current treatments make the search for effective therapies a real need. Different plant-derived essential oils (EOs) have shown antileishmanial effects, in particular from *Bixa orellana* L. (EO-Bo) and *Dysphania ambrosioides* (L.) Mosyakin & Clemants (EO-Da). In the present study, the EO-Bo and EO-Da, formulated in nanocochleates (EO-Bo-NC and EO-Da-NC, respectively), were evaluated in vitro and in vivo against *L. amazonensis*. The EO-Bo-NC and EO-Da-NC did not increase the in vitro inhibitory activity of the EOs, although the EO-Bo-NC showed reduced cytotoxic effects. In the animal model, both formulations (30 mg/kg/intralesional route/every 4 days/4 times) showed no deaths or weight loss greater than 10%. In the animal (mouse) model, EO-Bo-NC contributed to the control of infection (*p* < 0.05) in comparison with EO-Bo treatment, while the mice treated with EO-Da-NC exhibited larger lesions (*p* < 0.05) compared to those treated with EO-Da. The enhanced in vivo activity observed for EO-Bo-NC suggests that lipid-based nanoformulations like nanocochleates should be explored for their potential in the proper delivery of drugs, and in particular, the delivery of hydrophobic materials for effective cutaneous leishmaniasis treatment.

## 1. Introduction

Leishmaniasis is caused by zoonotic or anthroponotic transmission of protozoan parasites of the *Leishmania* genus (Trypanosomatidae). These maladies remain as neglected tropical diseases of major concern [1]. During a complex life cycle, close to 20 species of *Leishmania* parasites appear in two main forms: (i) the promastigote or extracellular and (ii) the amastigote or intracellular stages.

Leishmaniasis has been historically limited to endemic regions in the New (Central and South America) and the Old Worlds (Asia, Africa and southern Europe). Current estimates suggest that more than 10 million people are infected, with two million new cases and more than 20,000 deaths annually [2]. The diagnosis of these diseases is limited because of the broad spectrum of clinical manifestations, depending on species involved, and host immune response, which vary from cutaneous leishmaniasis (CL) to visceral leishmaniasis (VL) [3].

The antileishmanial treatments in current use include pentavalent antimonials, pentamidine, miltefosine, amphotericin B (AmB) and paramomycin, whose use is mainly limited in endemic regions, due to ineffectiveness, toxicity and high costs [4,5]. Furthermore, the absence of antileishmanial vaccines affirms the need to search for new treatment options for *Leishmania*-infected patients.

In this respect, the antileishmanial activity of several marine microalgae [6,7] and plant-derived products [8,9], including essential oils [10], has been evaluated. In previous studies, the essential oil (EO) from *Bixa orellana* L. (EO-Bo), Asteraceae, was highlighted for its inhibitory activity against intracellular amastigotes of *L. amazonensis*, lower cytotoxicity on the host cells and demonstrated significant therapeutic efficacy against experimental CL in mice [11]. In parallel, a serial of reports emphasized the potential activity and selectivity of EO from *Dysphania ambrosioides* (L.) Mosyakin & Clemants (syn. *Chenopodium ambrosioides* L.) (EO-Da), Amaranthaceae [12], as well as the capacity of EO-Da to prevent disease development in a mouse model of cutaneous leishmaniasis [13,14].

Nevertheless, several drawbacks, such as stability and solubility problems, have limited the use of EO-based treatments [15]. These issues can be solved by encapsulating the EOs into a lipid-based delivery system (LBDS), which allows delivering drugs of different chemical nature. In fact, the LBDSs are widely recommended for increasing the bioavailability of lipophilic compounds [16]. In this sense, the cochleates are LBDSs from the liposome family that look like cigar particles and can be obtained from the interaction of negatively charged phospholipids and divalent cations [17]. Cochleate preparations remain stable at 4 °C in a cation-containing buffer solution for about two years, and at least a year at room temperature as a lyophilized powder, allowing their oral or parenteral administration [18,19]. The sustained release achieved after administration of encochleated drugs contributes to increasing their circulation time and facilitates the drug transport directly to cellular and subcellular parasite locations [20,21]. Therefore, the present work was oriented to study the in vitro and in vivo antileishmanial effect of the EO-Bo and EO-Da formulated in nanocochleates (EO-Bo-NC and EO-Da-NC, respectively).

## 2. Results

### 2.1. Antileishmanial Activity and Cytotoxicity

The inhibitory activity on amastigotes of *L. amazonensis* and the cytotoxicity on peritoneal macrophages from BALB/c mice of nanocochleates in comparison with EOs were determined (Table 1). Concerning the anti-amastigote assay, both nanocochleate formulations lowered (*p* < 0.05) the in vitro leishmanicidal activity. Regarding the evaluation on peritoneal macrophages, the EO-Bo-NC showed less cytotoxicity with respect to EO-Bo (*p* < 0.05), while EO-Da-NC diminished the selectivity in comparison to EO-Da.

### 2.2. In Vivo Effect on Experimental Cutaneous Leishmaniasis

In the in vivo assay, treatment of mice with the EO-Bo-NC and EO-Da-NC (30 mg/kg/intralesional route/every 4 days/total of 4 doses) did not cause mortality or weight loss greater than 10% (Table 2).

**Figure 1:**
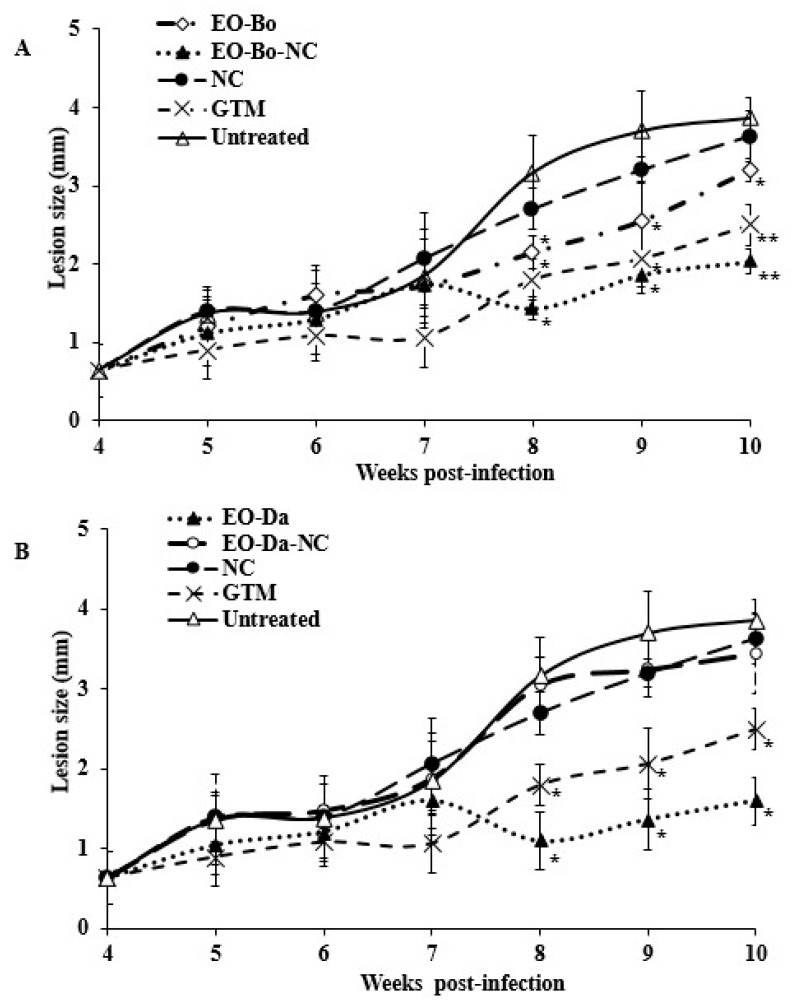
Effect of treatment with essential oil from *Bixa orellana* L. (**A**) and *Dysphania ambrosioides* (L.) Mosyakin & Clemants (**B**), in comparison with their nanocochleate formulations. BALB/c mice were infected with *L. amazonensis,* and 30 days post-infection the treatment was started with 4 doses by intralesional route at 30 mg/kg every four days. The results are expressed as the mean of lesion size in infected area ± standard deviation. EO-Bo: Essential oil from *B. orellana*; EO-Bo-NC: Essential oil from *B. orellana* formulated in nanocochleates. EO-Da: Essential oil from *D. ambrosioides*. EO-Da-NC: Essential oil from *D. ambrosioides* formulated in nanocochleates. NC: Empty nanocochleates. GTM: Glucantime^®^ used as reference drug. *: Displays statistical differences (*p* < 0.05) compared to untreated control and animals treated with NC. **: Displays statistical differences (*p* < 0.05) compared to animals treated with the respective EO.

The lesion size was measured in order to follow the disease evolution. With respect to EO-Bo (Figure 1A), the administration of EO-Bo-NC resulted in a smaller lesion size (*p* < 0.05), which was comparable to the effects of GTM (*p* > 0.05). However, mice that received EO-Da-NC showed larger lesion sizes (*p* < 0.05) compared with EO-Da and were similar (*p* > 0.05) to animals treated with NC and untreated mice (Figure 1B). Additionally, after administration of EO-Bo-NC, EO-Da-NC or NC, no successful cure was exhibited, considering that no differences (*p* > 0.05) with respect to parasite load were displayed (Log10 = 3.33–3.93 parasite/g).

## 3. Discussion

Despite the efforts, the absence of antileishmanial vaccines in the near future makes the discovering of effective chemotherapies an urgent need, especially for poor endemic regions [22]. Plant-derived compounds have accordingly come to represent a limitless source of chemical entities [23]. In particular, EOs have demonstrated a broad pharmacological spectrum [24] and several evidences have highlighted their leishmanicidal potential [9,10,25]. In particular, EOs from Cuban plants like *B. orellana* [11] and *D. ambrosioides* [12] have demonstrated their inhibitory activity on the in vitro growth of *L. amazonensis* and their ability to control the development of experimental CL in BALB/c mice. The aim of the present work was to study the in vitro and in vivo antileishmanial activity of nanocochleate formulations containing EO-Bo and EO-Da.

In order to demonstrate if the nanocochlear presentation was able to enhance the in vitro antileishmanial effect of EOs, the inhibitory activity was assayed. However, both nanocochleate formulations showed lower antileishmanial activity (*p* < 0.05). Considering that cochleates frequently delay the release of the encapsulated active principle [26], the action of EO-Bo-NC and EO-Da-NC may have been greatly compromised owing to the shorter exposure time routine used in these experiments.

However, with respect to the in vitro cytotoxic effects, incubation with EO-Bo-NC decreased the mortality of peritoneal macrophage compared to EO-Bo (*p* < 0.05). Previous works have demonstrated that, depending on the drug, the intracellular accumulation may vary [27]. The chemical profile of the EO products differs in the number and type of molecular stereochemical structures [24]. Therefore, despite the similar properties of the EOs, the different composition of them [11,14] in combination with cochleate precursors may have contributed to the contrary results observed on host cells for EO-Bo-NC with respect to EO-Da-NC.

Although several authors have highlighted the positive contributions of drug delivery systems against leishmaniasis [20,26], only a few works have focused on the use of cochleates. Nevertheless, some studies have been found in scientific literature, including: (i) the oral administration of AmB-loaded cochleates in *L. donovani* infected mice [28,29], (ii) the encochleation of AmB against *L. chagasi* [30] and (iii) the proteoliposome-derived cochleates in a model of CL by *L. major* [31]. Recently, our research group evaluated the antileishmanial activity of the EO from *Artemisia absinthium* L. presented in a stable, tolerable and efficacious nanocochleate formulation [32].

Promising results were obtained after administration of EO-Bo-NC preparations in the model of CL in BALB/c mice. This effect could be attributed to the ability of the cochleate membrane to fuse with cellular and subcellular membranes, which facilitated the arrival of encochleated material to the cytosol or to parasitophorous vacuoles to directly attack the pathogen [33]. In this process, several events can take place after administration of encochleated bioactive molecules, including: (i) drug accumulation, (ii) drug delayed release in a sustained manner, (iii) cochleate and (sub)cellular membrane fusion and (iv) cochleate clearance through the reticuloendothelial system. These events can contribute to directing the antileishmanial agent to the parasite host cell location, and to reducing unwanted side effects, which is desirable for effective treatment of infection due to *L. amazonensis* [20,24,30].

Nevertheless, the encochleation of a complex mixture of bioactive compounds like EOs may lead to different delivery rates for the compounds encapsulated. The LBDSs represent a reservoir, where the release of EO components can mainly happen through different ways: (i) dissolution, (ii) desorption and/or (iii) diffusion [34]. Some studies have also demonstrated that the EO release can take place in two phases. In the first phase, the molecules liberated are those located in the external wall, while in the second phase, the freed compounds are those present in the internal space [35]. In general, the EOs can include up to 60 components at different concentrations [24]. This fact may contribute to creating a release competition among them, which can super retard the delivery of drugs of concern like ishwarane in EO-Bo or ascaridole in EO-Da. In this sense, the multilamellar aspect of cochleates facilitates the separate inclusion of lipophilic and hydrophilic compounds in the internal spaces [36].

Infection with *L. amazonensis*, a major cause of CL in South America, is often associated with non-healing dermal lesions in people. In mice, the progress to ulcerated wounds is attributed to the extreme anergic pathogenicity that distinguished *L. amazonensis* amastigotes replication [37]. In the reasoning of effective and less toxic therapeutic alternatives, several ideas can be managed, including: (i) the increase of drug exposure time with minor doses, (ii) the drug transport to host cytosol and/or (iii) to parasitophorous vacuole. In the design formulation process, a possible way to fulfill these requirements contemplates the use of LBDS-like nanocochleates.

## 4. Materials and Methods

### 4.1. Essential Oils from B. orellana and D. ambrosioides

Fruits of *B. orellana* were collected on January 2015 in front of the Institute of Tropical Medicine Pedro Kouri, La Lisa, Havana (Cuba), and a specimen was deposited at the National Botanical Garden, Havana, Cuba, with a voucher specimen of NGB-9600288. Plants of *D. ambrosioides* were collected on July 2014 in Finca la Quiruvina”, Caimito, Artemisa (Cuba), and the specimen was deposited at the Experimental Station of Medicinal Plants “Dr. Juan Tomás Roig” with a specimen number of No. 4639.

The EO was obtained from fresh seeds and aerial parts (manually crushed) of *B. orellana* and *D. ambrosioides*, respectively, by hydrodistillation using Clevenger-type equipment over a 4-h period. Chemical constituents of obtained EO were corroborated by gas chromatography coupled with mass spectrometry (GC-MS) as previously reported [11,14], and compared. EO-Bo and EO-Da were hermetically sealed and stored in the Natural Product Collection of the Institute of Tropical Medicine Pedro Kouri under standard conditions (4 °C and darkness). For the biological assays, the EOs were dissolved in dimethyl sulfoxide (DMSO) at 20 mg/mL.

### 4.2. Reference Drug

Glucantime^®^ (GTM) from Rhône-Poulenc Rorer, Mexico, at a concentration of 30 mg/mL, was used as reference drug and dissolved in sterile saline solution to biological assays.

### 4.3. Nanocochleate Formulations

The dehydration–hydration modified method was employed to prepare nanocochleates (NC) containing EO-Bo or EO-Da, as previously described [32]. A suspension of opalescence aspect was obtained, in correspondence with the formation of small spherical vesicles like liposomes. The next step was filtering under sterile conditions through 0.2 μm, and enough CaCl_2_ was added to a final concentration of 10 mM in the formulation. For the preparation of empty cochleates (NC), a similar process was followed, but without the incorporation of the EO component. Lastly, the preparations were stored in amber flasks at 4 °C. In parallel, a sample of each NC was used to corroborate physical parameters, which was carried out by Electrophoretic Light Scattering, using Delsa™-Nano C (Beckman Coulter, Sweden), with a detector of 165 degrees. The particle size of EO-Bo-NC and EO-Da-NC varied between 52.3 and 96.1 nm. while that of the NC formulation was inferior to 40 nm. The polydispersion index of EO-Bo-NC and EO-Da-NC was between 0.325 and 0.335, whereas that of the NC preparation was between 0.44 and 0.52. Considering the difference of particle motility induced by different magnetic fields, the zeta potential for EO-Bo-NC and EO-Da-NC ranged from 40.4 to 41.2 mV and for NC between 31.1 and 31.3 mV. Finally, gas-chromatography with mass spectrometer detector demonstrated that no volatile components were identified in the supernatant of the NCs. From each formulation, a final volume of 3 mL was obtained, stored at 4 °C in an amber hermetically-sealed flask and used to carry out in vitro and in vivo studies.

### 4.4. Parasites

The strain MHOM/77BR/LTB0016 of *L. amazonensis,* donated by the Department of Immunology, Oswaldo Cruz Foundation (FIOCRUZ), Brazil, was used. Parasites were routinely isolated from an infected BALB/c mouse and directly cultivated in Schneider’s medium (Sigma-Aldrich, St. Louis, MO, USA) containing 10% heat-inactivated fetal bovine serum (HFBS, Sigma-Aldrich) and antibiotics (100 μg of streptomycin/mL and 100 U of penicillin/mL). Parasites were maintained as promastigotes at 26 °C with passages every 3 or 4 days and used between 5 and 10 in vitro passages to all experiments.

### 4.5. Animals

The animals were provided by The National Center of Laboratory Animals Production (CENPALAB), Cuba, and maintained under standard conditions. Forty-eight female BALB/c mice with a body weight from 20 to 22 g were used according to “Guideline on the Care and Use of Laboratory Animals”, which was approved by the Ethics Committee from the Institute of Tropical Medicine Pedro Kouri (CEI-IPK 14-12), Havana, Cuba.

### 4.6. Antiamastigote Assay

In order to evaluate the in vitro performance of EO-Bo-NC and EO-Da-NC, the antileishmanial activity facing intracellular amastigotes of *L. amazonensis* was evaluated according to Torres-Santos et al. [38] as follows. Briefly, peritoneal macrophages from normal BALB/c mice were obtained in RPMI medium (SIGMA, St. Louis, MO, USA) and antibiotics, seeded in 24-well Lab-Tek (Costar^®^, New York, NY, USA) plates and incubated at 37 °C and 5% CO_2_. After 2 h, free cells were removed, and a culture of stationary-phase *L. amazonensis* promastigotes was added at a 4:1 parasite/macrophage ratio in the same medium supplemented with 10% HFBS. The plate was incubated again for 4 h under the same conditions, and free parasites were discarded. After that, 1 mL of medium was added to the wells, and then 990 µL of medium containing 10 µL of products was added in the first wells. Serial dilutions 1:2 were performed. Untreated control, as well as cultures treated with NC or EO, were also included. The plate was incubated for an additional 48 h under the same conditions. The supernatant was then removed, the culture was washed, fixed with absolute methanol, stained with Giemsa and examined under a light microscope (Motic, Hong Kong, China) with immersion oil at 1000 ×. The infection rates were obtained by multiplying the percentage of infected macrophages by the number of amastigotes, counting in 25 macrophages per sample. Percent of inhibition of the infection rate in comparison to those of the controls was calculated. Two replicates were carried out to each product, and medium inhibitory concentration (IC_50_) was obtained from lineal regression curves. Results were expressed as medium of IC_50_ and standard deviation.

### 4.7. Cytotoxicity Assay

The cytotoxicity assay was carried out using the MTT method [39]. Briefly, peritoneal macrophages from BALB/c mice were collected, seeded at 3 × 10^5^ cells/mL and incubated in 96-well Lab-Tek (Greiner bio-one, Frichenhausen, Germany) at 5% CO_2_ and 37 °C. After 2 h, the medium was removed and 50 µL of fresh medium with 10% HFBS and antibiotics were added, with an additional 48 µL in the first wells and 2 µL of each product. An untreated control, as well as a culture treated with NC or EO alone, were also included. Then, five serial dilutions (1:2) were carried out, and an additional 50 µL of medium was added to each well. The plate was incubated at the same conditions for 48 h. Then, 15 µL of 3-[4,5-dimethylthiazol-2-yl]-2,5-diphenyltetrazolium bromide (MTT) (SIGMA, St. Louis, MO, USA) (5 mg/mL) was added to each well, and the plate was incubated under the same conditions. After 4 h, the formazan crystals were dissolved with 100 µL of DMSO, and the optical density was measured at 560 nm and 630 nm as a reference wavelength using a spectrophotometer (Sirio S Reader, 2.4-0, Seac and Radim Diagnostics, Calenzano, Italy). In each case, the medium cytotoxic concentration (CC_50_) was determined from lineal dose-response curves of three experiments. Results are expressed as means of CC_50_ with respective standard deviations.

### 4.8. In Vivo Studies

On day 0, female healthy BALB/c mice were infected by subcutaneous route in the right hind footpad with 5 × 10^6^ stationary-phase *L. amazonensis* promastigotes. After 4 weeks post-infection (p.i.), animals were randomly divided into seven groups of eight mice each, and the treatment was started. Products (EO-Bo, EO-Bo-NC, EO-Da, EO-Da-NC, NC and GTM) were administered by intralesional route at a dose of 30 mg/kg in a volume of 50 µL every 4 days to complete 4 administrations. An additional group did not receive any treatment, identified as untreated or control. Between 4 and 10 weeks p.i., deaths were registered daily, body weight was determined by group every 7 days, and the lesion size was determined weekly using a digital caliper, by measuring footpad swelling of the lesion diameter. Average lesion size for each group was calculated as the mean of the differences observed among infected and uninfected footpads. On week 6 and 10 p.i., three animals of each group were sacrificed by cervical dislocation, and parasite burden was determined, employing the culture microtitration method in 96-well plates according to Buffet et al. [40]. Briefly, a sample of the infected area in the footpad was excised, weighed and homogenized in 4 mL of Schneider’s medium. A serial four-fold dilution was prepared under sterile conditions in 96-well plates and was incubated at 26 °C for 7 days. Then, the plates were examined with an inverted microscope (Olympus, Japan). The final titer was defined as the last dilution for which the well contained at least one parasite. The parasite burden was calculated as the geometric mean of reciprocal titers from each duplicate/weight of homogenized cross-section × 400.

### 4.9. Statistical Analysis

Statistical differences, considered as *p* < 0.05, between the products in the in vitro assays, were determined using the Mann–Whitney test. In the mouse model, the lesion evolution and parasite load were processed by the Variance Analysis Test, accompanied by a Post Hoc Test (LDS test or planned comparison). The Statistica for Windows Program (Release 4.5, StatSoft, Inc. 1993) was used in all cases.

## 5. Conclusions

In conclusion, further studies aiming to explore the possible interactions occurring as a result of EO-Da encapsulation should be carried out in order to understand the moderated effects observed after EO-Da-NC treatment. Nevertheless, the present work demonstrated that the nanoencochleation of oily active principles like EO-Bo can be proposed for better control of leishmaniasis. Therefore, these encouraging findings may become a new starting point for the development of alternatives for CL treatment.

## Figures and Tables

**Table 1:** In vitro antileishmanial activity (IC_50_) and cytotoxic effect (CC_50_) of the essential oils from *Bixa orellana* L. and *Dysphania ambrosioides* (L.) Mosyakin & Clemants and their nanocochleate formulations.

Products	IC_50_^a^ ± SD^b^ (μg/mL)	CC_50_^c^ ± SD^b^ (μg/mL)	Selectivity Index
EO-Bo	8.5 ± 0.8	61.8 ± 5.9	7
EO-Bo-NC	15.4 ± 1.3*	94.6 ± 2.2*	6
EO-Da	4.9 ± 1.1	57.9 ± 3.7	12
EO-Da-NC	>60*	46.9 ± 4.4*	-
GTM	11.0 ± 3.4	>1500	>136
NC	~25% of infection at maximum volume tested.	~70% of mortality at maximum volume tested.	-

^a^ IC_50_: Concentration of product that caused 50% of inhibition growth. ^b^ SD: Standard deviation. ^c^ CC_50_: Concentration of product that caused 50% of macrophage mortality. EO-Bo: Essential oil from *B. orellana*. EO-Bo-NC: Essential oil from *B. orellana* formulated in nanocochleates. EO-Da: Essential oil from *D. ambrosioides*. EO-Da-NC: Essential oil from *D. ambrosioides* formulated in nanocochleates. GTM: Glucantime^®^. NC: Empty nanocochleates. *: Displays statistical differences (*p* < 0.05) compared to respective EO.

**Table 2:** Variations of body weight of animals infected with *L. amazonensis* and divided by groups of mice (n = 8) treated with the essential oils from *Bixa orellana* L. and *Dysphania ambrosioides* (L.) Mosyakin & Clemants and their nanocochleate formulations.

Group of Animals (Number of Animals)	Average of	Variation of Body Weight (%)^a^					
	Initial Weight (g)	5 w.p.i.	6 w.p.i.	7 w.p.i.	8 w.p.i.	9 w.p.i.	10 w.p.i.
EO-Bo (8)	19.2	+8.1	+9.4	+10.7	+12.7	+13.2	+16.4
EO-Bo-NC (8)	19.0	+3.5	+4.6	+7.9	+8.9	+9.3	+9.5
EO-Da (8)	19.3	+3.1	+3.7	+3.5	+7.3	+5.9	+11.8
EO-Da-NC (8)	19.5	+0.3	+3.1	+3.3	+10.7	+12.2	+11.2
NC (8)	19.7	−2.1	+0.3	+4.6	+8.0	+8.6	+8.6
GTM (8)	19.2	+2.9	+6.3	+5.5	+8.6	+8.4	+9.5
Untreated (8)	19.3	+4.0	+3.7	+5.8	+6.4	+6.3	+8.4

^a^ Positive number represents an increase of body weight, while negative number indicats a decrease of body weight, with respect to initial body weight at week 4 post-infection. EO-Bo: Essential oil from *B. orellana*. EO-Bo-NC: Essential oil from *B. orellana* formulated in nanocochleates. EO-Da: Essential oil from *D. ambrosioides*. EO-Da-NC: Essential oil from *D. ambrosioides* in nanocochleates. NC: Empty nanocochleates. GTM: Glucantime^®^ used as reference drug. w.p.i.: Weeks post infection.
